# Honoring Prof. Dr. Valentin A. Stonik for His Outstanding Contribution to Marine Natural Product Chemistry on the Occasion of His 80th Birthday

**DOI:** 10.3390/md22020056

**Published:** 2024-01-24

**Authors:** Pavel S. Dmitrenok, Natalia V. Ivanchina, Vladimir I. Kalinin

**Affiliations:** G.B. Elyakov Pacific Institute of Bioorganic Chemistry, Far Eastern Branch of the Russian Academy of Sciences, Pr. 100-Letya Vladivostoka 159, 690022 Vladivostok, Russia; paveldmt@piboc.dvo.ru (P.S.D.); ivanchina@piboc.dvo.ru (N.V.I.)

Marine natural products are a very structurally diverse group of preferably low-weight organic molecules. They began to be intensively studied in the 1960s. Except for their wide structural diversity, these metabolites are interesting as modulators of chemoecological interactions, possible active substances in medicinal and other useful preparations, and as taxonomic and food chain markers. Their biosynthesis and evolution of biosynthesis are also very interesting.

Prof. Dr. Sc. Valentin A. Stonik is one of the pioneers in this field; he began his impressive research career in marine natural products in the early 1970s and has continued his research activities in this field ever since. He was born in Vladivostok, Russia, on December 4, 1942 and graduated from the Department of Chemistry of the Far Eastern State University (Vladivostok) in 1965. Valentin A. Stonik received his PhD degree in Organic Chemistry in 1969 and Dr. Sc. degree in Bioorganic Chemistry: Chemistry of Natural and Physiologically Active Compounds in 1988. He became a Corresponding Member of the Russian Academy of Sciences in 1997 and a Full Member of the Russian Academy of Sciences (Academician) in 2003. He began his scientific activities in the synthesis of hydroacrydines and relative compounds [1]. Since 1970, he has worked in the G.B. Elyakov Pacific Institute of Bioorganic Chemistry, Far Eastern Branch of the Russian Academy of Sciences (PIBOC). Professor Stonik became Head of the Laboratory of Biosynthesis in 1976, Head of the Laboratory of the Chemistry of Marine Natural Products in 1985, Deputy Director of the Institute from 1990 to 2002, and Director from 2002 to 2017. Since 2018, he has been the Scientific Advisor of the Institute.

His general research scopes include the structure and properties of biphylic physiologically active natural products from marine invertebrates, especially echinoderms and sponges, and his specific interests include alkaloids [2], unusual lipids [3], isoprenoids [4], polyhydroxysteroids [5], glycosides of polyhydroxysteroids [6], steroidal [7] and triterpenoidal oligoglycosides [8], investigation of biological activities [9], biosynthesis [10], chemotaxonomy [11], and the chemical evolution of secondary metabolites [12]. In 1970–1990s, he led numerous scientific expeditions on the research vessels “Kallisto”, “Professor Bogorov”, and “Akademic Oparin” in different regions of the World Ocean. He is an author and co-author of more than 400 scientific articles in Russian and international journals, with more than 7500 citations, 4 monographs, and over 20 patents. He is a member of the Editorial Boards of the following Journals: *Marine Drugs*, *Natural Product Communications*, *Natural Product Letters*, *Russian Journal of Bioorganic Chemistry*, and others.

This Special Issue includes 12 contributions:

1. Silchenko, A.S.; Avilov, S.A.; Popov, R.S.; Dmitrenok, P.S.; Chingizova, E.A.; Grebnev, B.B.; Rasin, A.B.; Kalinin, V.I. Chilensosides E, F, and G—new tetrasulfated triterpene glycosides from the sea cucumber *Paracaudina chilensis* (Caudinidae, Molpadida): structures, activity, and biogenesis. *Mar. Drugs* **2023**, *21*, 114.

2. Menchinskaya, E.S.; Dyshlovoy, S.A.; Venz, S.; Jacobsen, C.; Hauschild, J.; Rohlfing, T.; Silchenko, A.S.; Avilov, S.A.; Balabanov, S.; Bokemeyer, C.; Aminin, D.L.; von Amsberg, G.; Honecker, F. Anticancer activity of the marine triterpene glycoside cucumarioside A_2_-2 in human prostate cancer cells. *Mar. Drugs* **2024**, *22*, 20.

3. Malyarenko, T.V.; Zakharenko, V.M.; Kicha, A.A.; Kuzmich, A.S.; Malyarenko, O.S.; Kalinovsky, A.I.; Popov, R.S.; Svetashev, V.I.; Ivanchina, N.V. New ceramides and cerebrosides from the deep-sea Far Eastern starfish *Ceramaster patagonicus*. *Mar. Drugs* **2022**, *20*, 641.

4. Popov, A.M.; Kozlovskaya, E.P.; Klimovich, A.A.; Rutckova, T.A.; Vakhrushev, A.I.; Hushpulian, D.M.; Gazaryan, I.G.; Makhankov, V.V.; Son, O.M.; Tekutyeva, L.A. Carotenoids from starfish *Patiria pectinifera*: therapeutic activity in models of inflammatory diseases. *Mar. Drugs* **2023**, *21*, 470.

5. Kikionis, S.; Papakyriakopoulou, P.; Mavrogiorgis, P.; Vasileva, E.A.; Mishchenko, N.P.; Fedoreyev, S.A.; Valsami, G.; Ioannou, E.; Roussis, V. Development of novel pharmaceutical forms of the marine bioactive pigment echinochrome A enabling alternative routes of administration. *Mar. Drugs* **2023**, 21, 250.

6. Dyshlovoy, S.A.; Fedorov, S.N.; Svetashev, V.I.; Makarieva, T.N.; Kalinovsky, A.I.; Moiseenko, O.P.; Krasokhin, V.B.; Shubina, L.K.; Guzii, A.G.; von Amsberg, G.; Stonik, V.A. 1-O-Alkylglycerol ethers from the marine sponge *Guitarra abbotti* and their cytotoxic activity. *Mar. Drugs* **2022**, *20*, 409.

7. Gartshore, C.J.; Wang, X.; Su, Y.; Molinski, T.F. Petrosamine revisited. Experimental and computational investigation of solvatochromism, tautomerism and free energy landscapes of a pyridoacridinium quaternary salt. *Mar. Drugs* **2023**, *21*, 446.

8. Dyshlovoy, S.A.; Shubina, L.K.; Makarieva, T.N.; Guzii, A.G.; Hauschild, J.; Strewinsky, N.; Berdyshev, D.V.; Kudryashova, E.K.; Menshov, A.S.; Popov, R.S.; Dmitrenok, P.S.; Graefen, M.; Bokemeyer, C.; von Amsberg, G. New guanidine alkaloids batzelladines O and P from the marine sponge *Monanchora pulchra* induce apoptosis and autophagy in prostate cancer cells. *Mar. Drugs* **2022**, *20*, 738.

9. Ivanov, I.A.; Siniavin, A.E.; Palikov, V.A.; Senko, D.A.; Shelukhina, I.V.; Epifanova, L.A.; Ojomoko, L.O.; Belukhina, S.Y.; Prokopev, N.A.; Landau, M.A.; Palikova, Y.A.; Kazakov, V.A.; Borozdina, N.A. Bervinova, A.V.; Dyachenko, I.A.; Kasheverov I.E.; Tsetlin V.I.; Kudryavtsev D.S. Analogs of 6-bromohypaphorine with increased agonist potency for α7 nicotinic receptor as anti-Inflammatory analgesic agents. *Mar. Drugs* **2023**, *21*, 368.

10. Usov, A.I.; Bilan, M.I.; Ustyuzhanina, N.E.; Nifantiev, N.E. Fucoidans of brown algae: comparison of sulfated polysaccharides from *Fucus vesiculosus* and *Ascophyllum nodosum*. *Mar. Drugs* **2022**, *20*, 638.

11. Davydova, V.N.; Krylova, N.V.; Iunikhina, O.V.; Volod’ko, A.V.; Pimenova, E.A.; Shchelkanov, M.Y.; Yermak, I.M. Physicochemical properties and antiherpetic activity of α-carrageenan complex with chitosan. *Mar. Drugs* **2023**, *21*, 238.

12. Solov’eva, T.F.; Bakholdina, S.I.; Naberezhnykh, G.A. Host defense proteins and peptides with lipopolysaccharide-binding activity from marine invertebrates and their therapeutic potential in Gram-negative sepsis. *Mar. Drugs* **2023**, *21*, 581.

Contributions 1–5 concern the metabolites from echnoderms, the representatives of the phylum Echinodermata, one of priority interests of Professor Valentin A. Stonik. The first contribution concerns the isolation of three new tetrasulfated triterpene glycosides, chilensosides E–G, isolated from the Far-Eastern sea cucumber *Paracaudina chilensis*. Their structures were elucidated using 2D NMR and ESIMS procedures. The compounds differ in carbohydrate chains; while chilensosides E and F are tetrasulfated pentaosides with the position of one of the sulfate groups at C-3 of the glucose residues which occupied third position in carbohydrate chains, chilensoside G is a tetrasulfated hexaoside. The isolation of tetrasulfated glycosides is rare.

The second contribution is dedicated to the study of the anticancer activity of triterpene glycoside cucumarioside A_2_-2 from the Far Eastern sea cucumber *Cucumaria japonica* in human castrate-resistant cancer. The authors found that the glycoside induced a G2/M-phase cell cycle arrest and caspase-dependent apoptosis via an intrinsic pathway. The glycoside also inhibited the formation of cancer cell colonies and growth in non-cytotoxic concentrations. A proteome analysis, using the 2D-PAGE technique and MALDI-MS followed by bioinformatical evaluation, revealed alterations in proteins involved in metastatic potential, invasion, and apoptosis. The regulation of keratin 81, CrkII, IL-1β, and cathepsin B was found. The results demonstrated the promising anticancer activity of cucumarioside A_2_-2 in a prostate cancer model.

The third contribution concerns the study of ceramides and cerebrosides from the deep-sea Far Eastern starfish *Ceramaster patagonicus.* The authors isolated three new ceramides and three new cerebrosides along with three known cerebrosides. The structures of the new compounds were elucidated using NMR and ESIMS procedures and the necessary chemical transformations. All the new cerebrosides have β-D-glucopyranose as a monosaccharide moiety. Most of the new compounds exhibited slight to moderate cytotoxic activity against human cancer cells (HT-29, SK-MEL-28, and MDA-MB-231) and normal embryonic kidney cells HEK293. They also inhibited the colony formation of MDA-MB-231.

The fourth contribution is dedicated to the study of the therapeutic activity of carotenoids from the Far Eastern starfish *Patiria pectinifera* in models of inflammatory diseases. The authors found that the carotenoids mixture isolated from the starfish *P. pectinifera* contains about 50% astaxanthin, 4–6% zeaxanthine and lutein, as well as free fatty acids and their glycerides. The complex exhibited anti-inflammatory, anti-allergic, and cancer-preventive activity without any toxicity at a dose of 500 mg/kg. The carotenoid mixture effectively improves the clinical picture of disease progression, as well as normalizing the cytokine profile and the antioxidant defense system in the in vivo animal models of inflammatory diseases, including skin carcinogenesis, allergic contact dermatitis, and systemic inflammation. The results show that the carotenoid complex from the starfish *P. pectinifera* may be effective for the treatment or prevention of different inflammations.

The fifth contribution concerns the development of novel pharmaceutical forms of the naphtaquinoid pigment echinochrome A, a characteristic metabolite of different sea urchins that possesses useful biological activities as an antioxidant. The authors incorporated echinochrome A isolated from *Diadema* sea urchins harvested near that island of Kastellorizo (Mediterranean Sea) in electrospun micro-/nanofibrous matrices composed of polycaprolactone and polyvinylpyrrolidone in different combinations. Ex vivo permeability studies using echinochrome A-loaded micro-/nanofibrous matrices showed an increased permeation of the pigment across the duodenum barrier. The results reveal that electrospun polymeric micro-/nanofibers may be promising carriers for new pharmaceutical formulations with controlled release, as well as increased echinochrome A stability and solubility useful for oral administration, and have the potential for the targeted delivery of echinochrome A.

The next three contributions cover articles concerning the metabolites of sponges that also are in the sphere of the primary scientific scopes of Professor Valentin A. Stonik. Contribution 6 concerns the investigation of 1-*O*-alkylglycerol ethers of marine sponge *Guitarra abbotti* and their cytotoxic activity. The authors determined the composition of the mixture of 1-*O*-alkylglycerol ethers using ^1^H and ^13^C NMR, GLC/MS, and chemical derivatization and found 6 new and 22 previously known 1-*O*-alkylglycerol ethers. The mixture reveals weak cytotoxic activity on HL-60, THP-1, DLD-1, HeLa, SKMEL-28, MDA-MB-231, and SNU C4 human cancer cells. Further cytotoxicity studies in JB6 P+ Cl41 cells bearing mutated MAP kinase genes revealed that JNK1 and ERK2 play a cytoprotective role in the cellular response to the 1-*O*-alkylglycerol ethers-induced cytotoxic effects.

Contribution 7 concerns the reinvestigation of the chemical and physical properties of a colored pyridoacridine alkaloid petrosamine isolated from the Belizean sponge, *Petrosia* sp., a potent inhibitor of acetylcholine esterase. The properties were investigated using spectroscopic and computational methods. The authors found that by analyzing petrosamine-free energy landscapes, pKa, and tautomerism, an accurate electronic depiction of the molecular structure is the di-keto form, with a net charge of q = +1, but this is not a dication (q = +2). Such molecular structure complements have been published in computational docking studies to define the contact points in the enzyme active site. This finding may improve the design of new acetylcholine esterase inhibitors based on such a molecular skeleton.

Contribution 8 is dedicated to the isolation and structural elucidation of two new guanidine alkaloids, batzelladines O and P, from the deep-water marine sponge *Monanchora pulchra* and studies of the induction of apoptosis and autophagy in prostate cancer cells by these substances. The structures of these alkaloids were elucidated using NMR spectroscopy, mass spectrometry, and ECD. The isolated substances revealed cytotoxic activity on human prostate cancer cells 22Rv1, PC3, as well as PC3-DR and inhibited colony formation and cancer cells survival. The alkaloids induced apoptosis detected by Western blotting as caspase-3 and PARP cleavage. The pro-survival autophagy, indicated as the upregulation of LC3B-II and downregulation of mTOR, was found to be treated by alkaloids cells. The use of the autophagy inhibitor 3-methyladenine in combination with alkaloids synergistically increased cytotoxic activity. In a combination of alkaloids with docetaxel, an additive effect was determined. The isolated new guanidine alkaloids may be promising drug candidates for the treatment of taxane-resistant prostate cancer.

The alkaloid 6-bromohypaphorine was isolated from the marine sponges *Pachymatisma johnstoni*, *Aplysina* sp., *Aplidium conicum*, the nudibranch *Hermissenda crassicornis* [13], and sea cucumber *Apostichopus japonicus* [14]. Such a wide distribution of this substance in marine invertebrates from so far taxa allows for its microbial origination to be suggested. L-6-bromohypaphorine acts as an agonist of the α7 nicotinic acetylcholine receptor (nAChR) involved in anti-inflammatory regulation [13]. The ninth contribution of this Special Issue concerns the synthesis of analogs of natural 6-bromohypaphorine with increased agonist potency for the α7 nicotinic receptor as anti-inflammatory analgesic agents. The authors used virtual screening to synthesize the analogs binding to the α7 nAChR molecular model. They synthesized fourteen analogs and tested them in vitro using a calcium fluorescence assay on the α7 nAChR expressed in neuro 2a cells. The synthesized methoxy ester of D-6-iodohypaphorine (6ID) revealed the highest potency being almost inactive toward α9α10 nAChR. The macrophages cytometry showed anti-inflammatory activity because of the decrease in the expression of TLR4 and increasing CD86, similar to the action of PNU282987, known as the selective α7 nAChR agonist. The methoxy ester of D-6-nitrohypaphorine revealed anti-oedemic and analgesic effects in an arthritis rat model. The tested compounds showed excellent tolerability with no acute in vivo toxicity. Thus, the combination of molecular modeling with a natural product-inspired drug design allowed for the preparation to be obtained with the desired activity of the chosen nAChR ligand. Hence, the isolation of the biological active natural product by the members of Professor Valentin A. Stonik’s group allows for the creation of new perspective candidates for the development of anti-inflammation preparations.

Contribution 10 is a review that concerns fucoidans of brown algae, namely the comparison of sulfated polysaccharides from *Fucus vesiculosus* and *Ascophyllum nodosum.* The authors noted that these biopolymers have many biological activities that may be used in practical applications. These two species from the family Phaeophyceae are known sources of commercial fucoidans. The authors conclude that fucoidans from these species are very complicated mixtures, and only fractions with carefully characterized structures prepared from both fucoidans may be used for drug development.

Contribution 11 concerns the obtaining of the κ-carrageenan polyelectrolyte complex with chitosan and studying its physicochemical properties and antiherpetic activity. The authors showed a two-fold increase in the antiherpetic activity (selective index) of the obtained polyelectrolyte complex compared to κ-carrageenan that may be caused by a change in the physicochemical characteristics of κ-carrageenan in the complex.

Contribution 12 is a review summarizing the data on lipopolysaccharide-binding proteins from marine invertebrates that may inhibit the toxic effects of bacterial lipopolysaccharides and are possible potential drugs for the treatment of lipopolysaccharide-induced sepsis. The structure of the proteins and peptides and their synthetic analogs, physicochemical properties, antimicrobial, and LPS-binding/neutralizing activity are described in detail. The problems which arise during the clinical trials of these substances are discussed.

Hence, all the contributions of this Special Issue revealed the wide scientific scopes and interests of Professor Valentin A. Stonik as a researcher and as the Director and Scientific Advisor of the G.B. Elyakov Pacific Institute of Bioorganic Chemistry.
